# Male-Female Differences in Upregulation of Vasoconstrictor Responses in Human Cerebral Arteries

**DOI:** 10.1371/journal.pone.0062698

**Published:** 2013-04-29

**Authors:** Hilda Ahnstedt, Lei Cao, Diana N. Krause, Karin Warfvinge, Hans Säveland, Ola G. Nilsson, Lars Edvinsson

**Affiliations:** 1 Division of Experimental Vascular Research, Department of Clinical Sciences, Lund University, Lund, Sweden; 2 Department of Pharmacology, School of Medicine, University of California Irvine, Irvine, California, United States of America; 3 Department of Neurosurgery, Lund University Hospital, Lund, Sweden; Emory University, United States of America

## Abstract

**Background and purpose:**

Male-female differences may significantly impact stroke prevention and treatment in men and women, however underlying mechanisms for sexual dimorphism in stroke are not understood. We previously found in males that cerebral ischemia upregulates contractile receptors in cerebral arteries, which is associated with lower blood flow. The present study investigates if cerebral arteries from men and women differ in cerebrovascular receptor upregulation.

**Experimental approach:**

Freshly obtained human cerebral arteries were placed in organ culture, an established model for studying receptor upregulation. 5-hydroxtryptamine type 1B (5-HT_1B_), angiotensin II type 1 (AT_1_) and endothelin-1 type A and B (ET_A_ and ET_B_) receptors were evaluated using wire myograph for contractile responses, real-time PCR for mRNA and immunohistochemistry for receptor expression.

**Key results:**

Vascular sensitivity to angiotensin II and endothelin-1 was markedly lower in cultured cerebral arteries from women as compared to men. ET_B_ receptor-mediated contraction occurred in male but not female arteries. Interestingly, there were similar upregulation in mRNA and expression of 5-HT_1B_, AT_1_, and ET_B_ receptors and in local expression of Ang II after organ culture.

**Conclusions and Implications:**

In spite of receptor upregulation after organ culture in both sexes, cerebral arteries from women were significantly less responsive to vasoconstrictors angiotensin II and endothelin-1 as compared to arteries from men. This suggests receptor coupling and/or signal transduction mechanisms involved in cerebrovascular contractility may be suppressed in females. This is the first study to demonstrate sex differences in the vascular function of human brain arteries.

## Introduction

Sexual dimorphism is observed in cerebral ischemia as demonstrated by a higher incidence of stroke in men than in women throughout much of the lifespan [1]–[3]. Sex differences also exist in the response to stroke treatments such as recombinant tissue plasminogen activator and aspirin [3]–[5].

The underlying mechanisms for male-female differences in stroke are not established, however, many studies point to a role for estrogen. After onset of menopause when estrogen levels decline, the incidence of cerebrovascular disease in women increases. Pre-clinical studies have indicated that estrogen is neuroprotective and reduces stroke infarct volume [6], [7]. Although sex steroid hormones are thought to be important contributors to sex differences in stroke, there is growing appreciation of other influences of biological sex, such as the role of sex chromosomes [3], [8]. For instance, cortical neurons from male and female embryos show marked differences in the response to ischemic-like insults [9].

We have discovered in human stroke patients that contractile G protein-coupled receptors (GPCRs) are upregulated in cerebral blood vessels associated with the infarct site [10]. Similarly after experimental stroke in rats, cerebrovascular receptor upregulation occurs in parallel with increases in contractile responses [11]. We hypothesize that increased expression of contractile GPCRs in vessels located in the penumbra or area at risk may reduce cerebral blood flow further after an ischemic stroke and thereby aggravate the tissue damage. The mechanisms associated with this phenomenon occur via transcription and activation of the MEK/ERK1/2 signal transduction pathway [11], [12]. Treatment with specific MEK/ERK1/2 inhibitors after experimental stroke suppresses upregulation of vasoconstrictor receptors and also reduces the brain infarct and improves neurological outcome [13], [14]. These studies suggest cerebrovascular receptor upregulation is correlated with stroke outcome.

The studies to date on cerebrovascular receptor upregulation have been conducted primarily on males or, in some cases, data from males and females have been combined. It is not known whether there are sex differences in this vascular response. To address this question in human cerebral arteries, we utilized an *in vitro* organ culture model to evoke ischemic-like contractile receptor changes in isolated arteries from human brain. We have established that organ culture, like experimental stroke, increases cerebrovascular expression of contractile GPCRs such as 5-hydroxytryptamine type 1B (5-HT_1B_), angiotensin II (Ang II) type 1 (AT_1_) and endothelin (ET-1) type B (ET_B_) receptors in the vascular smooth muscle cells (VSMCs) [11]. Organ culture is not a model for stroke *per se*, however it has been demonstrated to induce similar changes in vasoconstrictor responses, mRNA and protein expression of GPCRs, and intracellular Ca^2+^ levels as observed after focal ischemia [15]. Therefore, organ culture is a convenient method to mimic the changes in vasoconstrictor responses to provide knowledge about vascular receptor upregulation in different tissues and insight into underlying mechanisms. Supporting this approach, the MEK1/2 inhibitor U0126 and Raf inhibitor SB-386023 attenuated upregulation of ET_B_ receptors after organ culture *in vitro* [16], and after *in vivo* focal cerebral ischemia [13], [14] and subarachnoid hemorrhage [17], [18]. In these *in vivo* studies, attenuated vasoconstrictor responses were associated with increased global cerebral blood flow and improved neurology outcome.

In the present study, contractile responses and receptor upregulation in human arteries were measured after organ culture with the focus on possible male-female differences. We report similar increase in receptor mRNA and receptor expression in all vessels after organ culture, but the contractile responses differ markedly between the sexes, with female arteries being less responsive to vasoconstrictors Ang II and ET-1.

### Methods

This study was approved by the Regional Ethical Review Board in Lund, Sweden (LU-818-01), conforms to the principles outlined in the Declaration of Helsinki and subjects gave informed written consent.

### Tissue Collection

Human cerebral arteries (cortex) were obtained from patients undergoing neurological surgery for treatment of brain tumors or in a few cases severe epilepsy (4 males and 1 female). A total of n = 35 patients, 21 men and 14 women with mean age 55±3 and 50±6 years, respectively, were included in the study. None of the women were on estrogen therapy. Adjacent normal tissue was dissected during surgery, and arteries from this tissue were placed in cold sterile Dulbecco’s modified Eagle’s medium (DMEM, Gibco, Invitrogen, Carlsbad, CA). The outer diameter of obtained arteries ranged from 375 to 975 µm, with no differences between the female and male group. Cylindrical segments of 2 mm were either used directly, or cultured for 48 h in humidified 5% CO_2_ and air in serum-free DMEM supplemented with penicillin (100 U ml^−1^), streptomycin (100 µg ml^−1^) and amphotericin B (0.25 µg ml^−1^) (Invitrogen) with change of medium after 24 h, prior to further experiments. Most often the material obtained from each patient was sufficient to be divided and analyzed with two of the three methods described below.

### *In vitro* Pharmacology

Contractile responses of human cerebral arteries after organ culture were examined in a Mulvany Halpern myograph (Danish Myo Technology A/S, Aarhus, Denmark) by recording isometric tension as described previously [12]. Each arterial segment was mounted in a 5 ml temperature-controlled tissue bath (37°C) on two parallel stainless steel wires (40 µm) inserted into the lumen. Each segment was stretched to 90% of the normal internal circumference, which is the size the vessel would have if it were relaxed under a transmural pressure of 100 mm Hg [19]. The contraction induced by 63.5 mM K^+^ was used as a reference for contractile capacity [20]. To check for endothelial function, segments were pre-contracted with 5-hydroxytryptamine (5-HT, 0.3 µM) for 3 min followed by application of carbachol (10 µM) for 2 min.

Concentration-response curves were obtained by cumulative application of 5-CT (5-HT_1B_ receptor agonist [21], 10^−11^ to 3·10^−5 ^M), Ang II (AT_1_ and AT_2_ receptor agonist, 10^−12^ to 3·10^−6^ M) and ET-1 (ET_A_ and ET_B_ receptor agonist, 10^−14^ to 3·10^−7^ M). The effect of the selective ET_B_ agonist, sarafotoxin S6c, was initially tested in a few patient samples but no contractile responses were observed in agreement with a previous study [22]. Instead, the dual ET_A_ and ET_B_ receptor agonist ET-1 was used to generate a biphasic concentration-response curve, and we assessed the initial high-affinity phase which corresponds to ET_B_ receptor-mediated contraction [23]. The effects of indomethacin (10 µM) and L-NG-nitroarginine methyl ester (L-NAME, 100 µM), synthesis inhibitors of prostaglandins and nitric oxide (NO), respectively, were also examined in selected samples by continuous presence of the inhibitors throughout the experiment. Receptor nomenclature stated in the present study confirms to guidelines for receptors and channels [24].

### RNA Extraction and Real-time PCR

RNA extraction and real-time PCR was performed as described earlier [25]. Briefly, RNA was isolated using an RNeasy Mini kit (Qiagen GmbH, Hilden, Germany) and transcribed to cDNA using the TaqMan Reverse Transcription Reagents (Applied Biosystems, Foster city, CA, USA). Individual assays using cDNA and primers for the 5-HT_1B_, AT_1_ and ET_B_ receptors were performed with the SYBR® Green kit on a GeneAmp 7300 (Applied Biosystems) and normalized to housekeeping gene β-actin. Primers used were designed as follows: 5-HT_1B_ forward: 5′- AAA TCC CCA TCC CTG AAG GGT ATGA-3′; 5-HT_1B_ reverse: 5′- AGC AGC AGT GTG GGC TGA GT-3′; AT_1_ forward: 5′- GGA TGG TTC TCA GAG AGA GTA CAT-3′; AT_1_ reverse: 5′- CCT GCC CTC TTG TAC CTG TTG-3′; ET_B_ forward: 5′- GAT ACG ACA ACT TCC GCT CCA-3′; ET_B_ reverse: 5′- GTC CAC GAT GAG GAC AAT GAG-3′; β-actin forward: 5′- GTA GCC ATC CAG GCT GTG TTG-3′; β-actin reverse: 5′- TGC CAG TGG TAC GAC CAG AG-3′.

### Immunohistochemistry

Sections (10 µm) from paraformaldehyde or acetone fixed arteries were incubated with rabbit anti-human 5-HT_1B_ 1∶100 (#ab13896, Abcam, Cambridge, UK), rabbit anti-human AT_1_ 1:100 (#sc-1173, Santa Cruz Biotechnology, Santa Cruz, CA), rabbit anti-human Ang II 1∶250 (#NBP1-30027, Novus Biologicals, Littleton, CO), sheep anti-human ET_B_ 1∶100 (#ALX-210-506A-C250, Enzo, Lausen, Switzerland), mouse anti-human ET-1 1:250 (#ab2786, Abcam) or mouse anti-human phosphospecific ERK1/2 1:200 (#ab50011, Abcam) and then with appropriate secondary antibodies as described earlier [12]. Omission of primary antibody served as negative control, which resulted in no staining except for autofluorescence in the lamina elastica interna. Furthermore, control experiments were performed by incubating the AT_1_ antibody with its blocking peptide (1∶5 in weight) prior to the immunohistochemistry experiment. Immunoreactivity was visualized using an epifluorescence microscope (Nikon 80i; Tokyo, Japan).

### Analysis and Statistics

Data are expressed as mean ± standard error of the mean (SEM) and n refers to the number of patients. Statistical analyses were performed with Mann-Whitney (two groups) and Kruskal-Wallis test followed by Dunn’s multiple comparison test (three groups) non-parametric test where P<0.05 was considered significant. The concentration-response curves for each agonist were analyzed with extra sum-of-squares F test to determine if the curves were statistically different in male and female arteries.

#### In vitro pharmacology

Contractile responses of human cerebral arteries are expressed as percent of the contraction induced by 63.5 mM K^+^. The maximum contraction elicited by an agonist is referred to as the E_MAX_, and pEC_50_ represents the negative logarithm of the agonist concentration that produced half the maximum response. For the ET-1 biphasic concentration-response curve, E_MAX (1)_ and pEC_50 (1)_ describe the high-affinity phase, and E_MAX (2)_ and pEC_50 (2)_ describe the low-affinity phase. One to three artery segments per patient were mounted in the wire myograph, and a mean value were calculated to represent each patient.

#### Real-time PCR

The amount of mRNA in each sample was calculated relative the amount of β-actin mRNA in the same sample by the formula *X_0_/R_0_* = 2*^CtR-CtX^*, where *X_0_* is the original amount of target mRNA, *R_0_* is the original amount of β-actin mRNA, *CtX* is the *C_T_* value for the target and *C_T_R* is the *C_T_* value for β-actin. The *C_T_* values refer to the number of PCR cycles performed for each PCR product in a sample at a specific time point. Four female and eight male patient samples were used (each sample in duplicate). The resulting values were examined using Grubb’s test and outliers were removed.

#### Immunohistochemistry

Each experiment was repeated two to three times to ensure reproducibility. Qualitative assessment of the immunohistochemical staining was evaluated by two human readers, blinded to the study details and with extensive expertise in the field. Comparison of immunoreactivity in fresh and organ cultured tissue was always performed within tissue from the same patient. Five female and six male samples were evaluated in each group.

Additionally, measurement of the fluorescence intensity in the smooth muscle cell layer of each artery section was performed in a blinded manner using the Image J software. This was performed to further evaluate the immunoreactivity and, in addition, strengthen the qualitative assessment made by the human reader. The mean intensities (arbitrary units) of male and female arteries incubated for 0 h and 48 h of organ culture are presented. Because of the comparative studies, immunoreactivity to individual receptors was visualized with the same microscope settings during the same day.

## Results

### *In vitro* Pharmacology

Contractile responses of human cerebral arteries were examined after organ culture by *in vitro* pharmacology methods using a wire myograph. K^+^-induced contractions did not differ significantly between male and female arteries (Table 1). Carbachol (10 µmol/L) was used to test for endothelium integrity in arteries pre-contracted with 5-HT (0.3 µmol/L); however after organ culture, there was no acetylcholine receptor-mediated relaxation in either male or female cerebral arteries. Furthermore, a few experiments were performed with continuous blockade of prostaglandin and NO synthesis by indomethacin and L-NAME which had no effect on the vasoconstrictor responses. Together, these results indicate a lack of functional endothelium in cultured arteries.

**Table 1:** Contractile responses to 5-CT, Ang II, and ET-1.

			*Sigmoidal curve*		*Biphasic curve*			
	*n*	*K^+^ (mN)*	*E_MAX_ (%)*	*pEC_50_*	*E_MAX(1)_ (%)*	*pEC_50(1)_*	*E_MAX(2)_ (%)*	*pEC_50(2)_*
*5-CT*								
Males	9	8.6±1.2	54.1±10.0	7.0±0.3				
Females	6	10.7±1.0	46.0±4.8	6.8±0.3				
*Ang II*								
Males	9	8.5±1.1	47.4±5.4	9.9±0.3^a^				
Females	6	10.7±1.0	45.2±10.4	9.0±0.3				
*ET-1*								
Males	8	8.5±1.1			26.9±12.2^b^	11.8±0.3	121.3±9.2	8.9±0.1
Females	6	10.7±1.0			6.0±4.9	**	127.8±6.9	8.6±0.2

Responses were characterized by E_MAX_, expressed as percent of 63.5 mM K^+^-induced contraction, and pEC_50_. Values are represented as mean ± SEM, with *n* representing the number of patients. Statistical analyses were performed using the non-parametric Mann-Whitney’s test.

a P = 0.09,

b P = 0.08 compared to females.

** Response too low to accurately calculate pEC_50_. 5-CT –5-carboxamidotryptamine, Ang II – angiotensin II, ET-1– endothelin-1.

#### Contractile responses to 5-carboxamidotryptamine

**Figure 1:**
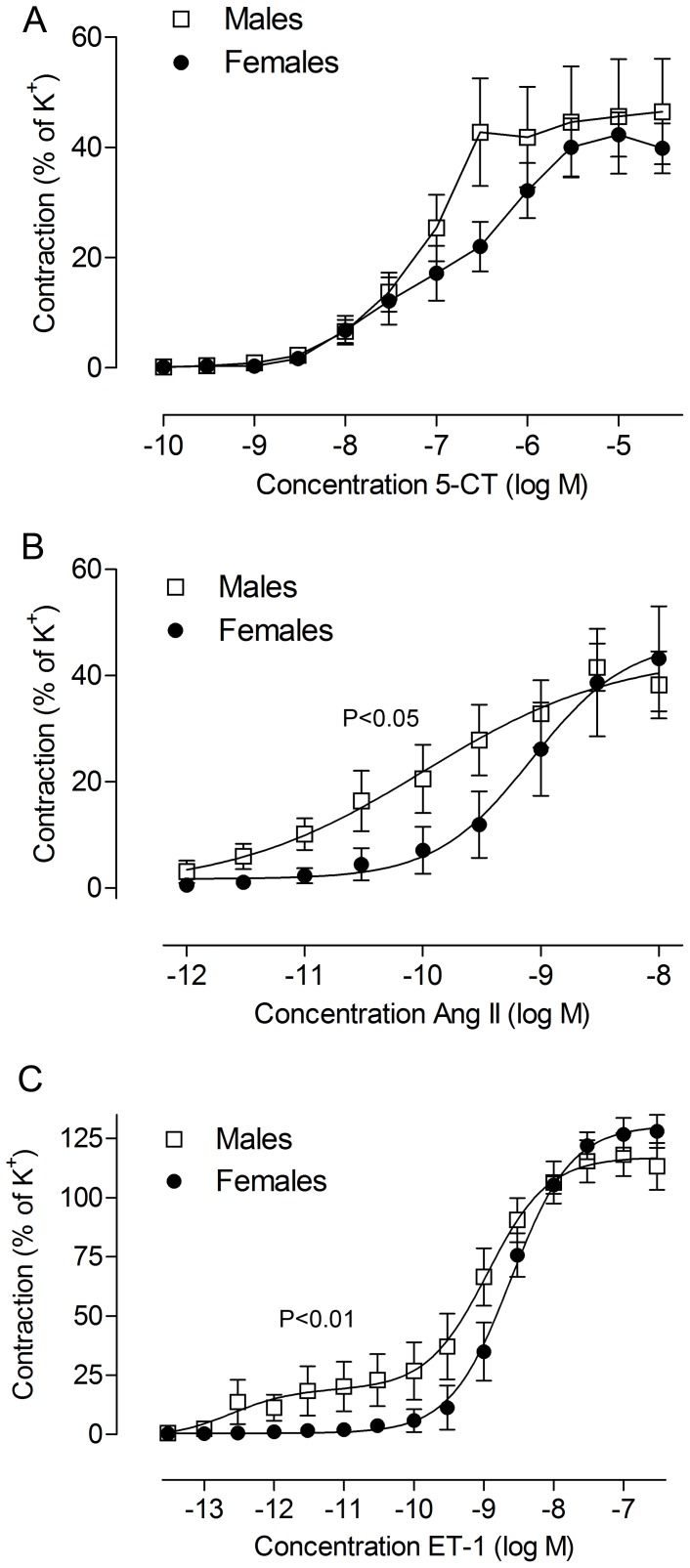
Male-female differences in contractile responses of human cerebral arteries subjected to 48 h organ culture. Concentration-response curves to (A) 5-CT, (B) Ang II and (C) ET-1 are illustrated. Data are expressed as % of the contraction to K^+^ (63.5 mM) in the same tissue. Each point represents mean ± SEM; females, n = 6 and males n = 8 to 9. The concentration-response curves for each agonist were analyzed with extra sum-of-squares F test to determine if the curves were statistically different between male and female arteries (P-values are presented above the curves). Exact values and statistical significance for individual parameters such as E_MAX_ and pEC_50_ are found in Table 1.

5-HT_1B_ receptor-mediated contraction was studied by cumulative application of increasing concentrations of the agonist 5-CT. 5-CT induced contractile responses that followed a monophasic concentration-response curve (Figure 1A). No significant differences between male and female cerebral arteries were observed in 5-HT_1B_ receptor-mediated contraction (Figure 1A, Table 1).

#### Contractile responses to angiotensin II

Ang II induced a concentration-dependent contraction at lower concentrations (10^−12^ to 10^−8^ mol/L, Figure 1B) and dilatation at higher concentrations (3·10^−8^ to 3·10^−6 ^mol/L, data not shown). These responses occurred in both male and female arteries after organ culture, but the male arteries were more sensitive to Ang II resulting in concentration-response curves that were significantly different (P<0.05, Figure 1B). pEC_50_ for Ang II-mediated contraction was higher for males compared to females, as shown by a concentration-response curve further to the left for males (P = 0.09, Figure 1B, Table 1). The maximum contraction to Ang II was not different between male and female arteries.

#### Contractile responses to endothelin-1

In male cerebral arteries exposed to organ culture, application of ET-1 elicited a biphasic concentration-dependent response indicating the presence of both ET_B_ receptors (high affinity) and ET_A_ receptors (low affinity). However, no ET_B_ receptor-mediated contraction was observed in three of the male patient samples. In all female arteries, ET-1 elicited a monophasic response demonstrating presence of ET_A_ receptors, but weak or no ET_B_ receptor-mediated contraction (Figure 1C). This was shown by a difference in the maximum contraction of the first phase in the ET-1 contraction (P = 0.08, Figure 1C, Table 1) and statistically different curves (P<0.01, Figure 1C). ET_A_ receptor-mediated contraction was not significantly different in male and female arteries. In addition, we tested the selective ET_B_ receptor agonist sarafotoxin S6c, which did not induce any vasoconstrictor responses, in agreement with earlier observations in fresh and cultured human cerebral arteries [12], [22], [26].

### Age

The pEC_50_ values for 5-CT and Ang II, and the E_MAX(1)_ for ET-1 were plotted against age of the patient to examine possible correlations between age and vasomotor reactivity. Linear regression analyses showed that the slopes were not significantly different from zero, except for Ang II. The linear regression for Ang II in female cerebral arteries (P<0.05, *r^2^* = 0.63) had a slope of -0.04±0.01 indicating a minor decrease in pEC_50_ with increasing age.

### Real-time PCR

**Figure 2:**
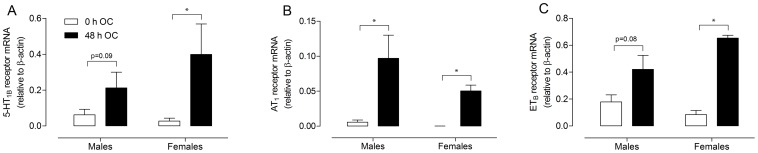
Effect of organ culture on mRNA levels of cerebrovascular receptors in human cerebral arteries. mRNA expression for (A) 5-HT_1B_ receptors, (B) AT_1_ receptors and (C) ET_B_ receptors before (0 h OC) and after organ culture (48 h OC) are illustrated. Each bar represents mean ± SEM; females, n = 4 and males n = 8; *P<0.05.

The effect of organ culture on 5-HT_1B_, AT_1_ and ET_B_ receptor mRNA levels was evaluated by real-time PCR of arterial segments taken before and after culture. 5-HT_1B_ receptor mRNA was significantly increased in females after organ culture (P<0.05, Figure 2A) while the set statistical level of significance was not reached in males (P = 0.09). However, previous data have shown this in diseased stroke patients [10]. AT_1_ receptor mRNA levels were markedly increased in both male and female cerebral arteries (P<0.05, Figure 2B). Incubation of cerebral arteries for 48 h increased ET_B_ receptor mRNA expression in females (P<0.05, Figure 2C). This difference was not statistically significant in males although a trend was observed (P = 0.08). In the present study no significant male-female differences were observed for receptor mRNA levels in either fresh (non-incubated) arteries, or in arteries after organ culture (48 h OC). However, non-existent sex differences in receptor mRNA after organ cannot be concluded from the present study due to large variations and low n numbers (males n = 8, females n = 4). To significantly detect a comparatively small difference as between male and female arteries after organ as in 5-HT_1B_ and AT_1_ receptor mRNA culture with a power of 50% n = 35 and n = 28 sample size is estimated (GraphPad Statmate 2.0, GraphPad Software Inc., La Jolla, CA, USA).

### Immunohistochemistry

Htx-eosin staining was performed on all specimens. No morphological differences were observed between non-incubated (fresh) and incubated arteries or between male and female arteries, which is in agreement with earlier data [12].

#### Upregulation of G-protein coupled receptors and angiotensin II in vascular smooth muscle

**Figure 3:**
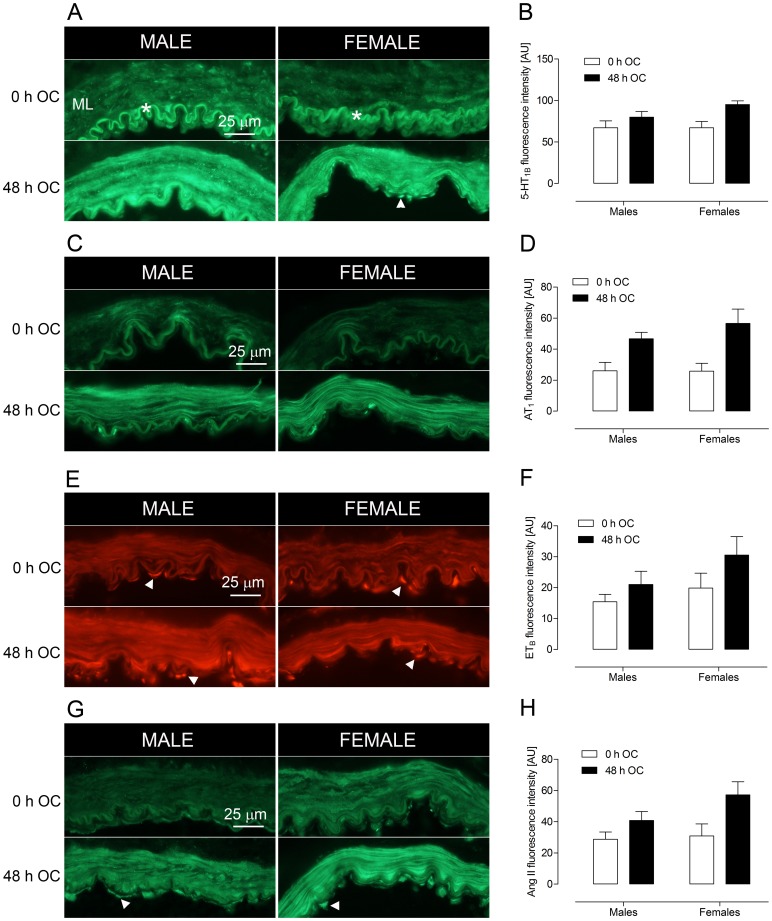
Upregulation of contractile receptors and Ang II in human cerebral arteries after organ culture (OC). The smooth muscle layers (ML, medial layer) show a dramatic increase in immunofluorescent staining of (A, B) 5-HT_1B_ receptors, (C, D) AT_1_ receptors, (E, F) ET_B_ receptors and (G, H) Ang II after 48 h of culture (48 h OC) as compared to non-incubated arteries (0 h OC). Fluorescent intensity measurements (arbitrary units, AU) were performed in the medial layer (ML), and are represented as mean ± SEM (right panel). Ang II, 5-HT_1B_ and ET_B_ immunofluorescence can also be seen in endothelial cells (when they are present, arrowheads). Autofluorescence in the internal lamina elastica is indicated by the asterisk.

Protein expression of 5-HT_1B_, AT_1_ and ET_B_ receptors and Ang II were examined by indirect immunohistochemistry of both fresh arteries and arteries incubated for 48 h. 5-HT_1B_, AT_1_ and ET_B_ receptor immunoreactivity was markedly increased in VSMCs of incubated (48 h OC) male and female arteries compared to fresh arteries (Figure 3A, 3C, 3E). Interestingly, female and male arteries showed increase of receptor immunoreactivity after organ culture and no obvious sex differences in receptor immunoreactivity was detected. This was surprising in light of the differences in contractility that were observed (Figure 1). The expression of Ang II was also examined. An increase in immunoreactivity was observed in the medial layer of both male and female arteries after 48 h of organ culture (Figure 3G). In addition, fluorescence intensity measurements in the smooth muscle cell layer showed increased immunoreactivity in both male and female cerebral arteries after organ culture (Figure 3B, 3D, 3F, 3H). ET-1 immunoreactivity in arteries from male and female patients showed high inter-individual differences. Consequently, no conclusive result could be obtained regarding ET-1 expression in fresh and incubated arteries in these patients.

#### Localization of G-protein coupled receptors and angiotensin II in endothelial cells

Immunoreactivity to 5-HT_1B_ and ET_B_ receptors and Ang II was found not only in the smooth muscle, but also in endothelial cells (Figure 3, arrowheads). This endothelial localization was observed in both male and female arteries, and in fresh and incubated arteries. However, endothelial immunoreactivity could not be found in all patients, most likely due to loss of endothelial cells during handling, organ culture or cryosectioning.

#### Activation of ERK1/2

**Figure 4:**
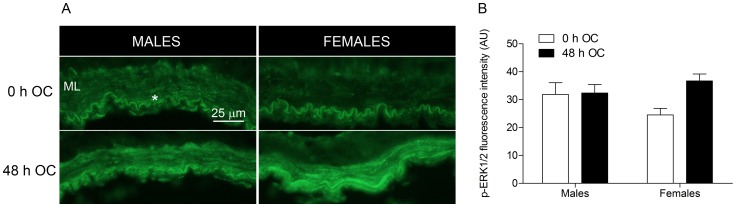
Phosphorylated ERK1/2 (p-ERK1/2) expression in male and female cerebral arteries after organ culture (OC). (A) Representative images of p-ERK1/2 in the medial layer (ML) of human cerebral arteries before (0 h OC) and after organ culture (48 h OC). Similar expression of p-ERK1/2 was observed in male arteries, while an increased immunoreactivity was observed in female arteries after organ culture. (B) Fluorescence intensity measurement (arbitrary units, AU) of p-ERK1/2 immunohistochemical staining in the ML.

In order to achieve more knowledge about underlying mechanisms we investigated activation of the Raf/MEK/ERK1/2 signaling pathway known to have an important role in receptor upregulation [12]. Phosphorylated ERK1/2 (p-ERK1/2) expression in fresh and incubated male and female cerebral arteries was examined by immunohistochemistry. Immunoreactivity to p-ERK1/2 was not observed in VSMCs of non-incubated female cerebral arteries (Figure 4A). However, we observed a robust increase in p-ERK1/2 expression after organ culture, substantiated by fluorescence intensity measurements in the smooth muscle cell layer (Figure 4B). We observed similar p-ERK1/2 expression in male cerebral arteries before and after organ culture (Figure 4A), also indicated by the intensity measurements (Figure 4B).

## Discussion

This study shows for the first time significant sex differences in vasoconstrictor responses of human cerebral arteries. Cerebral arteries from women were less responsive to Ang II and ET-1 as compared to arteries from men. These differences were observed 48 h after arteries were placed in organ culture. Although it is not a model of stroke *per se*, organ culture induces upregulation of cerebrovascular contractile receptors, modeling what is found after both clinical and experimental stroke [11]. Increased vasoconstriction via these receptors is hypothesized to exacerbate ischemic damage after stroke. Consequently, sex differences in this response would plausibly contribute to known male-female differences in stroke [1], [3].

The most striking finding in comparing male and female human cerebral arteries after organ culture was that, in spite of upregulation of receptor expression in all vessels, female arteries were much less responsive than male arteries to vasoconstrictor effects of Ang II and ET-1. In contrast, no male-female differences were detected in contractile responses to the 5-HT_1B_ agonist 5-CT. In addition, we found no differences in relaxant responses to carbachol in 5-HT pre-contracted arteries. We also observed no sex differences in 5-HT_1B_, AT_1_ and ET_B_ receptor mRNA or receptor expression in either fresh or cultured human cerebral arteries, assessed by real-time PCR and immunohistochemistry. Similar observations were made in cerebral arteries from patients that died of stroke; there were no obvious sex differences in the expression of these receptors [10]. However in stroke patients of both sexes, the levels of cerebrovascular receptor mRNA and protein were increased as compared to arteries of control subjects that died of extracranial events, cardiogenic insufficiency or myocardial infarction [10]. Similar to arteries of stroke patients, the human arteries in the present study had increased receptor expression after organ culture, but no sex differences were found. A quantitative method to assess receptor protein levels is desired to fully conclude no male-female differences in receptor expression after organ culture. However, this was not possible in the present study due to limited access and small amount of tissue obtained from each patient.

In an attempt to gain more knowledge about underlying mechanisms we examined the expression of p-ERK1/2 since an important role of the Raf/MEK/ERK1/2 pathway in receptor upregulation has been established earlier in human cerebral arteries [12]. Our findings indicate a robust increase in p-ERK1/2 expression in females after 48 h of organ culture, while male cerebral arteries at this point demonstrated similar expression as non-incubated arteries. Activation of ERK1/2 has been shown to occur early after initiating organ culture [16] and possibly ERK1/2 have already been activated and returned to baseline at 48 h in male arteries.

The advantage of the organ culture model is that we can assess contractile function as well as mechanisms involved in receptor expression in human cerebral arteries. Using this model, we found a sex difference in Ang II potency after culture; the pEC_50_ was 9.9 in males compared to 9 in females. Interestingly, the E_MAX_ for Ang II was increased to a similar degree (45–47%) in cultured arteries from men and women as compared to the E_MAX_ (12%) measured for Ang II in freshly isolated human cerebral arteries [pooled male and female tissue; 27]. The increase in E_MAX_ likely reflects the upregulation of AT_1_ receptor expression that occurred in both male and female human arteries.

Sex differences in contractile responses to Ang II have been observed in mouse middle cerebral arteries [28], and in the aorta and mesenteric arteries from spontaneously hypertensive rats [29]. In all cases male arteries showed a much stronger response to Ang II. In spontaneous hypertensive rats, the sex differences in contraction correlated with differences in AT_1_ receptor expression. However, in mouse cerebral arteries, the levels of AT_1_ receptors did not differ between males and females even though contractile responses to Ang II were greater in male arteries. These findings are similar to what we observed in human cerebral arteries after organ culture. The data suggest that coupling of AT_1_ receptors to contraction is less efficient in female cerebral arteries.

Experimental stroke is associated with increased levels of brain Ang II [30], [31]. In the present study, Ang II levels also increased in both male and female human cerebral arteries after 48 h organ culture. Ang II immunoreactivity was localized to both VSMCs and endothelial cells. Although there were no sex differences in Ang II expression, our potency data from the culture model suggest that cerebral arteries in men would be more sensitive to any stroke-related rise in Ang II.

The present study also showed sex differences in the contractile responses of human cerebral arteries to ET-1. After organ culture, a biphasic concentration-response curve to ET-1 was seen in male arteries, consistent with activation of both ET_A_ and ET_B_ receptors. However in female human cerebral arteries, ET-1 produced only a monophasic concentration-response curve reflective of ET_A_ receptor-mediated contraction. Thus male and female arteries had similar ET_A_-mediated responses, but female arteries showed little or no response via the high affinity ET_B_ receptor. It should be noted that three of the male patient samples demonstrated no or weak ET_B_ receptor-specific vasoconstriction. Although the reason for these observations cannot be explained, medication history and age of these patient samples were not any different from the patient samples showing strong ET_B_ receptor-mediated vasoconstriction.

We previously found in rat male arteries that ET_B_-mediated contraction upregulates after organ culture, but female arteries were not studied [32]. However, sex differences in vascular ET-1 responses have been reported for deoxycorticosterone acetate-salt hypertensive rats. With hypertension, male rat aortae exhibited increased responsiveness to low concentrations of ET-1, an effect blocked by the ET_B_ antagonist BQ788 [33]. Furthermore, the ET_B_ receptor selective agonist IRL-1620 increased aortic contraction in male deoxycorticosterone acetate-salt hypertensive rats, but not in females. This study suggested that the sex differences reflect changes in ET_A_/ET_B_ receptor expression but not in calcium handling mechanisms [34]. However, in our cultured human cerebral arteries, sex differences in ET_B_ contraction cannot be explained by differences in receptor expression. Results from immunohistochemistry and real-time PCR showed upregulation of ET_B_ receptor expression and mRNA in both sexes; however ET_B_ receptor expression in females did not result in contraction. The lack of response at the high affinity ET_B_ receptor suggests female cerebral arteries would be less sensitive to ET-1 during an ischemic episode.

We have no information about the hormonal status of the patients included in the present study. The men and women had a mean age of 55 and 50 years, respectively, indicating that many of the women likely had declining or cessation of ovarian function associated with menopause. However, all patient samples that were used for contractile studies were from post-menopausal women well beyond the average menopausal years, indicating that estrogen did not account for the observed male-female differences in vascular function after organ culture. Nevertheless, organizational changes from life-long exposure of female sex hormones cannot be excluded. Although the patient samples used for real-time PCR and immunohistochemistry was mixed from estimated pre-menopausal, menopausal and post-menopausal women, a clear increase in receptor expression and Ang II was observed independent on hormonal stage and age.

We do not know if and to what extent sex hormones contributed to the differences observed between males and females, but a number of studies indicate cerebral arteries can be influenced by estrogen, progesterone and testosterone [35]. Estrogen decreases, while testosterone increases, vascular tone in rodent cerebral arteries, and these effects are endothelium-dependent [36]–[38]. In the present study, however, endothelial influences were absent in the cultured human arteries, as evidenced by the lack of carbachol relaxation, a standard test for functional endothelium in cerebral arteries. The present study was designed to investigate receptor upregulation in the smooth muscle cells, and possible sex differences in endothelium-derived responses were not within the scope of this paper. Endothelium-independent mechanisms of estrogen have been reported in studies where estrogen treatment reduced the contractile responses to Ang II, histamine and 5-HT in human internal mammary arteries [39] and in rat aorta [40]. The effect in the latter study was independent of functional endothelium [40] as was the effect of estrogen on Ca^2+^ channel inhibition in cerebral arteries [41]. In experimental stoke, estrogen is neuroprotective and decreases infarct volume [6], [7]. A possible role of estrogen in decreasing contractile responses to Ang II and ET-1 in female arteries after stroke or organ culture remains to be investigated.

Interestingly, we observed that arteries from women who were more elderly (73–75 yrs) also were less responsive than arteries from men, suggesting these sex differences persist after menopause. The lower incidence of stroke in women compared to men is seen until the age of 85 years [3]. Thus mechanisms underlying male-female differences in human arteries may not require continued presence of hormone, but instead result from influences of sex chromosomes and/or organizational effects of sex hormones that commit tissues to a male or female phenotype [3].

### Conclusions

Marked male-female differences were found in the contractile responses to Ang II and ET-1 in human cerebral arteries after organ culture. Cerebral arteries from women were less responsive than those of men. These differences do not appear to involve sex-dependent differences in receptor expression since upregulation of cerebrovascular AT_1_ and ET_B_ receptors was observed in both sexes after organ culture, a model of cerebrovascular receptor changes after stroke. The mechanisms underlying decreased responsiveness in females remain to be determined but may involve changes in receptor coupling or signal transduction. Decreased vasoconstrictor sensitivity in female cerebral arteries may help to explain male-female differences that exist in cerebrovascular disease.

### Limitations of the Study

The supply of human material is limited, and was obtained during neurosurgery for treatment of tumors or epilepsy. Although adjacent non-cancerous or seizure-producing tissue was carefully dissected out by the neurosurgeons, effects on adjacent tissue cannot be excluded. The present patient material had some obvious short comings such as variation in subject age, differences in medication, associated disorders and variation in outer diameter of the received cerebral arteries. Therefore, fresh (non-incubated) and organ cultured arterial segments were prepared for each patient so that all comparisons could be made within the same patient. There wasn’t any correlation between existing drugs and the receptor-mediated responses that were observed.
